# Gene signatures of 6-methyladenine regulators in women with lung adenocarcinoma and development of a risk scoring system: a retrospective study using the cancer genome atlas database

**DOI:** 10.18632/aging.202364

**Published:** 2021-01-10

**Authors:** Chundi Gao, Jing Zhuang, Huayao Li, Cun Liu, Chao Zhou, Lijuan Liu, Fubin Feng, Changgang Sun

**Affiliations:** 1 College of First Clinical Medicine, Shandong University of Traditional Chinese Medicine, Jinan 250014, Shandong, PR China; 2 Departmen of Oncology, Weifang Traditional Chinese Hospital, Weifang 261041, Shandong, PR China; 3 College of Basic Medical Sciences, Shandong University of Traditional Chinese Medicine, Jinan 250014, Shandong, PR China; 4 Cancer and Immunology Institute, Shandong University of Traditional Chinese Medicine, Jinan, Shandong, PR China

**Keywords:** female lung adenocarcinoma, 6-methyladenine regulators, prognostic risk scoring system, Cox regression analysis, copy number variation

## Abstract

Although the emergence of new treatments has improved the prognosis of women with lung adenocarcinoma (LUAD), the emergence of drug resistance limits their clinical efficacy. Therefore, there is an urgent need to identify new targets and develop a risk scoring system to evaluate the prognosis of patients. 6-methyladenine (M6A), as the most common methyl modification in RNA modification, its clinicopathological features, diagnosis and prognostic value in lung cancer, especially in LUAD remain to be discussed. We analyzed the clinical and sequencing data of the female LUAD cohort from The Cancer Genome Atlas (TCGA), evaluated the expression profiles of 16 M6A regulation-related genes in the cohort and the relationships between genetic changes and clinical characteristics, developed an M6A-related risk scoring system using Cox analysis. Finally, the copy number variations (CNVs) of the related genes in the samples were analyzed and verified using the cBioPortal platform. Compared with other clinical factors, this risk scoring system showed a higher predictive sensitivity and specificity. The M6A-related risk scoring system developed in this study may help to improve the screening of female patients at high risk of LUAD and provides important theoretical bioinformatics support for evaluating the prognosis of such patients.

## INTRODUCTION

Lung cancer is the most common malignant tumor and the main cause of cancer-related mortality worldwide. Non-small cell lung cancer (NSCLC) accounts for about 80% of all lung cancers, including adenocarcinoma, squamous cell carcinoma and large cell carcinoma. The incidence of lung adenocarcinoma (LUAD), the most common subtype of NSCLC, is increasing year by year [1]. Different from lung squamous cell carcinoma, LUAD is more likely to occur in women and non-smokers and is a heterogeneous disease characterized by a high rate of gene mutations [2]. Although, targeted therapy and immunotherapy have greatly improved the outcomes and prognosis of patients with LUAD in recent years, the emergence of drug resistance is still inevitable, and the long-term survival rate is still low [3, 4]. Especially for a large proportion of female patients with lung adenocarcinoma, there is still a lack of targeted risk prediction system. Therefore, it is still an arduous task to evaluate and improve the prognosis of female patients with LUAD. In recent years, with the deepening of tumor research, it is recognized that the expression of oncogene depends not only on the gene itself, but also on epigenetic modification without changing the gene sequence [5, 6].

With the extensive study of epigenetic changes in tumor progression, researchers finally focus on DNA, RNA and histone modifications. As a post-transcriptional regulation strategy, RNA modification occurs widely in all kinds of RNA [7], especially in mRNAs. Among the RNA modifications, 6-methyladenine (M6A) is the most common methyl modification in mRNAs that regulate RNA splicing, translocation, stability, and transformation. The level of M6A is dynamically regulated by a methyltransferase (encoder), a binding protein (reader), and a demethylase (decoder), so, it is a dynamic and reversible process. Related studies have shown that changes in M6A regulatory genes could promote the progression of breast cancer, liver cancer, and hematological malignant tumors by inducing the formation of cancer stem cells and their abnormal differentiation [8–10]. It has also been reported that miR-33a can inhibit the proliferation of non-small cell lung cancer (NSCLC) cells by targeting M6A regulatory factor *METTL3* mRNA 3'UTR binding site [11]. Through the description of the genetic variation of M6A regulatory factor in lung adenocarcinoma, it was found that *FTO* and *YTHDF3* mutations were related to the decrease of overall survival rate [12]. A series of studies have shown that once the components involved in the regulation of M6A modification have been lost or abnormal, it would lead to abnormal physiological functions such as cell differentiation, and gene expression would be abnormally activated or inhibited. Whether the level of M6A can be reversed or enhanced to improve the clinical efficacy of tumor patients is worthy of further exploration.

Various types of genomic changes, including CNV, played an important role in promoting the occurrence and development of cancer, and were also key factors leading to genetic and epigenetic abnormalities [13]. Through the study of 270individuals, Redon and colleagues constructed the first generation CNV map of the human genome, and identified 1447 copy number variable regions in these populations, accounting for 12% of the genome. Further exploration found that the nucleotide content of CNV region was more than single nucleotide polymorphism, so CNV played an more important role in genetic diversity [14]. CNV, the amplification or deletion of DNA caused by genomic instability, can be used as an important therapeutic target, as it is related to drug resistance and tumor biology in many cancers, such as breast cancer and non-small cell lung cancer (NSCLC) [15, 16]. Li et al found that two subgroups of NSCLC, LUAD and lung squamous cell carcinoma, can be distinguished by comparing their CNV patterns [17]. Therefore, the full analysis of the clinicopathological features and prognostic value of the key M6A regulatory factors and CNV through the sample data was very important for the further exploration of LUAD.

As a powerful resource of genomic data on various cancers, The Cancer Genome Atlas (TCGA) is very helpful in studying the molecular mechanisms of cancer and identifying prognostic markers. For example, Shukla et al. obtained a four-gene signature that significantly stratified the overall survival rate for LUAD through the analysis of EGFR wild type and EGFR mutation subgroups of LUAD [18]. In this study, we analyzed the clinical and sequencing data on the female LUAD cohort from TCGA, evaluated the expression profiles of 16 M6A regulatory genes in the cohort and the association between genetic changes and clinical characteristics, and developed an M6A-related risk scoring system to evaluate the prognosis of female patients with LUAD. The copy number variations (CNVs) of the related genes in the samples were analyzed and verified using the cBioPortal platform. Notably, compared with other clinical factors, this risk scoring system showed a higher predictive sensitivity and specificity.

## RESULTS

### Extraction and analysis of M6A-related genes

**Figure 1:**
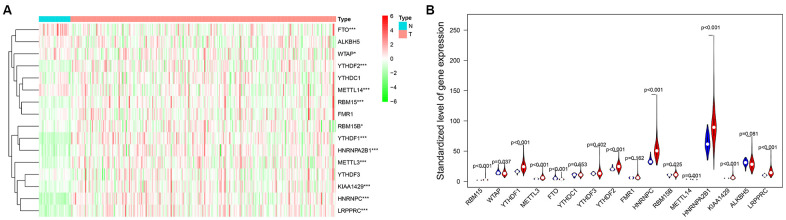
****(**A**) The heat map of differential expression of M6A-related genes in normal samples and female lung adenocarcinoma samples. The color from green to red shows a trend from low expression to high expression. (**B**) The violin diagram of differential expression of M6A-related genes in normal samples and female lung adenocarcinoma samples. The Y axis is the standardized level of gene expression. Red represents high expression, blue represents low expression. P<0.05 as the statistical cutoff value.

M6A-related protein is an important regulator of tumorigenesis, and its expression level often directly determines the pathological process of tumor. In this study, we systematically analyzed the expression of 16 widely reported M6A RNA regulatory factors in female LUAD and normal tissues. These 16 widely reported M6A related genes were not only selected in lung cancer related articles, but also selected as key genes for RNA M6A methylation in other tumor types [19–22]. By analyzing the mRNA expression in samples from female LUAD patients in TCGA, 16 M6A-related genes were screened, and their differential expression in normal and female LUAD samples were analyzed using the edger package. With P<0.05 as the cutoff value, there were some differences in the expression of these 16 genes between the normal and female LUAD samples, especially for *LRPPR*, *YTHDF1*, *HNRNPC*, *METTL3*, *METTL14*, *RBM15*, *HNRNPA2B1*, *FTO*, *KIAA1429*, and *YTHDF2* (Figure 1).

### Derivation of the risk scoring system for female LUAD

The risk scoring system for female LUAD was developed based on the analysis of the 16 M6A-related genes. Firstly, we incorporate 16 M6A-related genes (*METTL3*, *METTL14*, *WTAP*, *KIAA1429*, *RBM15*, *RBM15B*, *YTHDC1*, *YTHDF1*, *YTHDF2*, *YTHDF3*, *HNRNPC*, *HNRNPA2B1*, *FMR1*, *LRPPRC*, *ALKBH5* and *FTO*.) into the multivariate model. Then, based on the code cox <-coxph (Surv (futime, fustat) ~., data = rt), multivariate Cox analysis was performed to output five genes (*METTL3*, *YTHDF1*, *HNRNPC*, *HNRNPA2B1*, *FTO*) and correlation coefficients that can be used to build a risk scoring system (Table 1). The risk score for each sample was determined according to the coefficient analysis of the five genes, as follows:

**Table 1:** The specific baseline clinicopathological characteristics of 266 female LUAD samples.

**Clinical character**	**266 female LUAD samples with prognostic information**
Age	
<= 60 years	89
> 60 years	177
Stage	
I	156
II	52
III	43
IV	12
Unknown	3
Pathologic T stage	
T1-2	236
T3-4	28
Unknown	2
Pathologic N stage	
N0-1	219
N2-3	38
Unknown	9
Pathologic M stage	
M0	167
M1	11
Unknown	88
Survival time	
<= 1 years	57
1 years <	160
<= 3 years	
3 years <	32
<= 5 years	
> 5 years	17

Risk score = (- 0.4356 × *METTL3* expression) + (- 0.5791 × *YTHDF1* expression) + (1.1551 × *HNRNPC* expression) + (0.5820 × *HNRNPA2B1* expression) + (0.6329 × *FTO* expression)

**Figure 2:**
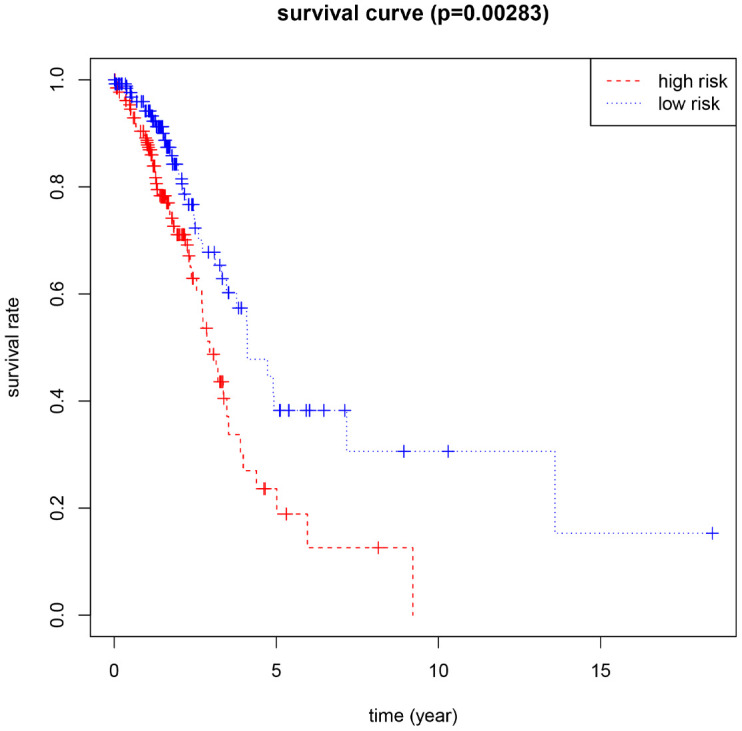
**Kaplan-Meier for the risk scoring system based on 5 M6A-related genes (P<0.05 as the statistical cutoff value).**

Using the median risk score as the cut-off value, 266 female LUAD samples with prognostic information were divided into high-risk and low-risk groups, and the overall survival (OS) of the low-risk group was significantly longer than that of the high-risk group (Figure 2). In addition, *HNRNPC*, *HNRNPA2B1*, and *FTO* showed positive coefficients, indicating that these genes were closely related to the high prognostic risk of female LUAD, that is, their higher expression levels corresponds to shorter OS. However, *METTL3* and *YTHDF1* showed negative coefficients, indicating that the lower their expression, the shorter the OS.

### Correlation between risk score and clinical traits

**Figure 3:**
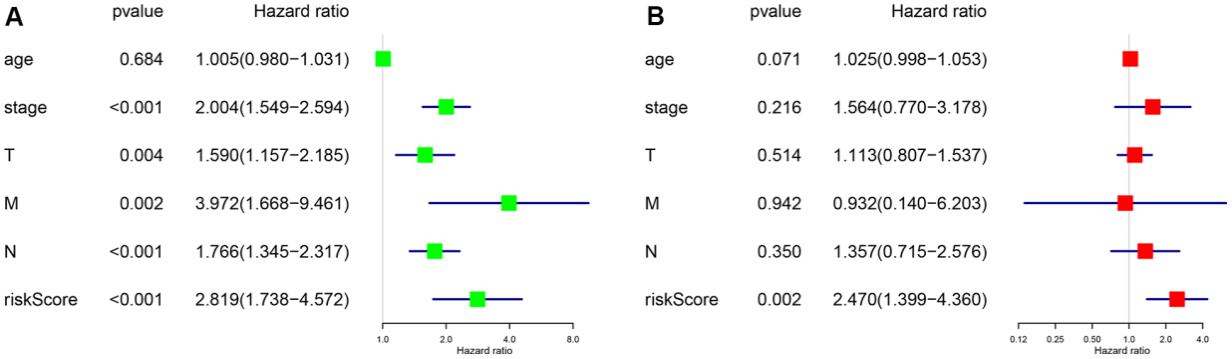
**P <0.05 as the statistical cutoff value.** (**A**) Univariate analysis of risk scores and clinical traits in female lung adenocarcinoma samples from TCGA database. (**B**) Multivariate analysis of risk scores and clinical traits in female lung adenocarcinoma samples from TCGA database.

**Figure 4:**
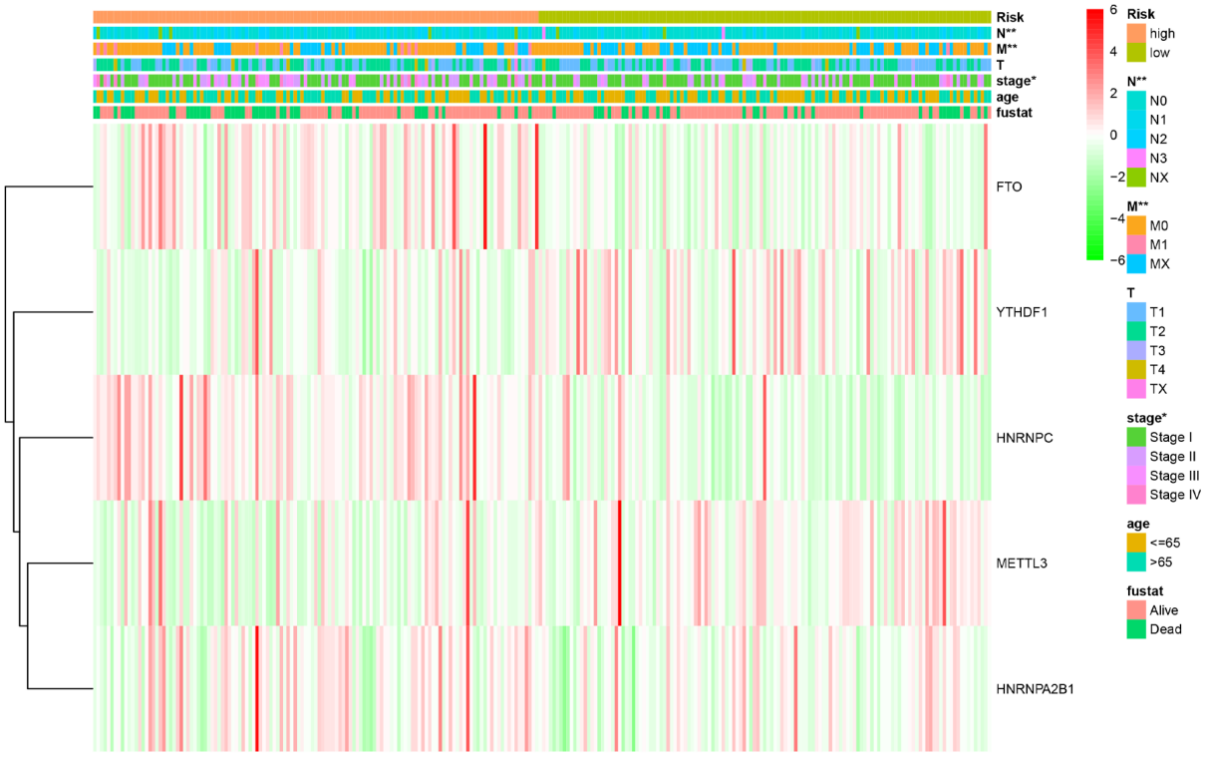
The relationship between the risk score and different clinical traits (* represents p < 0.05, ** represents p < 0.01, and *** represents p < 0.001).

To further evaluate the predictive performance of the risk scoring system that we developed, we included relevant clinical factors in this study (Table 2). Univariate Cox analysis showed that risk score, stage, and N stage were all closely related to OS in female patients with LUAD. Multivariate Cox analysis showed that with p<0.05 as the cutoff value, risk score could be used as an independent prognostic factor for evaluating patient survival time compared with clinical characteristics such as age and stage (Figure 3). Whether through univariate or multivariate Cox analysis, the risk scoring system could effectively evaluate the prognosis of female patients with LUAD, which further demonstrates the value of this model. In addition, we evaluated the relationship between the risk score and different clinical traits, and found that staging and N and M stages were all correlated with the risk score (P <0.05). And the higher the stage, N, and M stages, the higher the risk score, which is consistent with the current clinical prognosis, and further illustrates the accuracy of the risk scoring system we constructed. (Figure 4).

**Table 2:** Multivariate Cox analysis of M6A related genes in female patients with lung adenocarcinoma.

**Gene**	**coef**	**exp(coef)**	**se(coef)**	**z**
YTHDF1	-0.5791	0.5604	0.3024	-1.915
METTL3	-0.4356	0.6469	0.2118	-2.056
FTO	0.6329	1.8830	0.3016	2.098
HNRNPC	1.1551	3.1743	0.3335	3.463
HNRNPA2B1	0.5820	1.7897	0.3126	1.862

*Coef : the regression coefficient, exp (coef) : the risk ratio, se (coef) : the standard error, z : the Wald statistical value.

### CNVs of M6A-related genes in the risk scoring system

**Figure 5:**
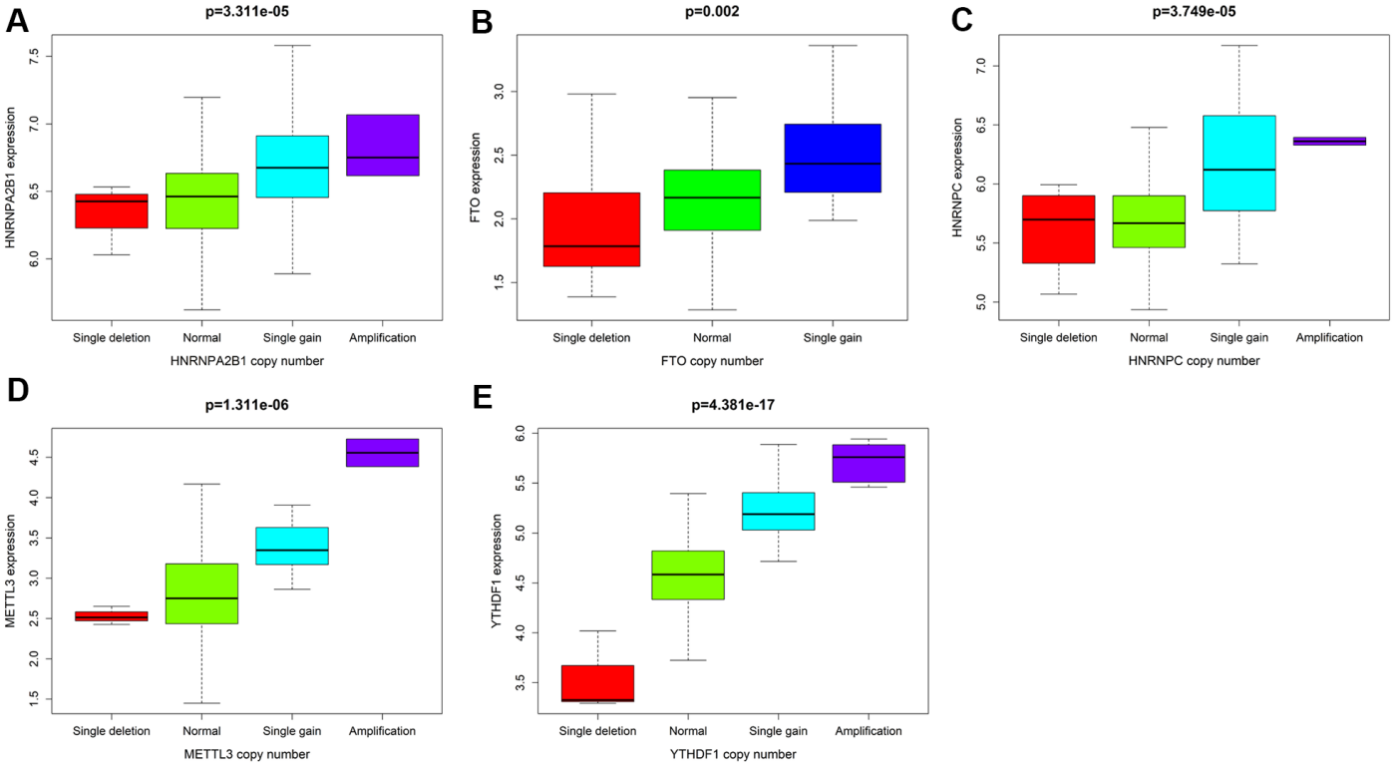
**With P<0.05 as the cutoff value, the correlation analysis between CNV mutations and the expression of 5 M6A-related genes.** (**A**) *HNRNPA2B1* (**B**) *FTO* (**C**) *HNRNPC* (**D**) *METTL3* (**E**) *YTHDF1*.

**Figure 6:**
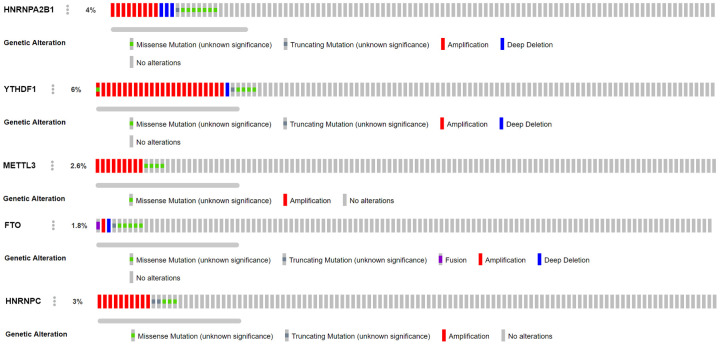
**Analysis of CNV mutations in 5 M6A-related genes in lung adenocarcinoma data of cBioportal platform.** Red represents an amplification in the number of copies, blue represents a deletion in the number of copies, green represents missense mutation, and gray represents truncating mutation.

Combined with the CNV data analysis of female LUAD samples, it was found that compared with normal samples, the five M6A-related genes used to construct the risk scoring system in female LUAD samples were prone to CNV (Table 3), and there was a correlation between CNV and the expression of these genes, and they all showed a trend of increasing expression with increase in their copy numbers (Figure 5). Analysis of the cBioportal platform-related data also revealed the proportion of CNVs of these genes, which further verified the results obtained in the TCGA data analysis (Figure 6). Pathway enrichment analysis showed that *METTL3*, *YTHDF1*, *HNRNPC*, *HNRNPA2B1*, and *FTO* could participate in genetic information processing, which is related to the biological process of mRNAs.

**Table 3:** The main chromosomes and related sites where CNV occurs in the 5 M6A-related genes in the risk scoring system.

**Chromosome**	**chromStart**	**chromEnd**	**Gene**
chr7	26189927	26201529	HNRNPA2B1
chr14	21209136	21269494	HNRNPC
chr14	21498133	21511375	METTL3
chr16	53703963	54121941	FTO
chr20	63195429	63216234	YTHDF1

## DISCUSSION

Lung adenocarcinoma (LUAD) is the most common pathological subtype of non-small cell lung cancer (NSCLC), with an average 5-year survival rate of patients with LUAD is only 18%. Different from lung squamous cell carcinoma, LUAD is more likely to occur in women and non-smokers. Although great progress has been made in neoadjuvant radiotherapy, chemotherapy, molecular targeted therapy and immunotherapy methods, and greatly increase clinical expectations for the successful treatment of malignant diseases [23–26], local recurrence and distant metastasis caused by tumor heterogeneity and drug resistance are still threatening the lives of patients, resulting in a low long-term survival rate [27, 28]. There is an urgent need to identify effective biomarkers to improve treatment strategies and predict the prognosis of patients.

Cancer is a complex genetic and epigenetic disease, which involves mutations and dysregulation of oncogenes and tumor suppressor genes [29, 30]. Among them, epigenetic modifications can participate in the occurrence and progression of tumors by inhibiting the expression of tumor suppressor genes and enhancing that of oncogenes [31, 32]. The inherently reversible epigenetic changes make them a potential target for drug therapy [33]. They showed great potential in clinical personalized treatment and in prolonging the survival time of patients. M6A played a key role in different diseases including cancer and the expression was related to the activation or inhibition of many carcinogenic pathways [34]. For example, Xinyao Lin et al found that M6A could regulate the progression of epithelial-mesenchymal transformation *in vitro* and *in vivo* and the M6A modification of mRNAs increases in cancer cells were an important step in cancer cell metastasis. In addition, they also found that the up-regulated M6A regulatory factors *METTL3* and *YTHDF1* were important factors leading to the poor prognosis of hepatocellular carcinoma [35]. Qiang Wang et al found that high expression of M6A regulatory factors *METTL3* in gastric cancer could play a carcinogenic role by regulating different targets or pathways and was closely related to the poor prognosis of patients with gastric cancer [36].

M6A regulatory factors are closely related to the activation and inhibition of cancer-related pathways and are potentially useful prognostic markers. In different types of cancer, M6A regulatory factors have been shown to undergo a wide range of genetic changes, including mutations and CNVs [37]. In this study, 16 widely reported M6A RNA regulatory factors were selected. Through multivariate COX analysis, five M6A-related gene markers, namely HNRNPC, HNRNPA2B1, FTO, METTL3, and YTHDF1, with high predictive ability were determined as a prognostic model for female LUAD. We divided 266 female LUAD samples into two high-risk and the low-risk groups according to the median risk scores of the five gene markers, and confirmed a significant difference in OS between the two groups. Combined with the CNV data analysis of female LUAD samples, it was found that compared with normal samples, the five M6A-related genes used to construct the risk scoring system in female LUAD samples were prone to CNV. Further, we observed a correlation between the CNVs and the corresponding gene expression. Our analyses emphasize the importance of M6A modulators in cancer development and lay the foundation for the development of novel therapeutic strategies based on RNA methylation.

*HNRNPC*, *YTHDF1*, and *HNRNPA2B1* are all involved in the formation of M6A as readers. Among them, *HNRNPC* can regulate the stability and translation levels of bound mRNA molecules [38], while M6A can promote the binding of mRNAs and *HNRNPC* through the "m6A-switch" mechanism, thus regulating mRNA splicing and affecting the expression of the corresponding proteins [39]. Huang et al found that *HNRNPC* can promote cell proliferation, migration, and invasion [40], which was closely related to the occurrence and progression of cancer and was an important M6A regulatory factor that caused poor prognosis of patients. *YTHDF1* can recognize and bind the M6A methylation of mRNA, thus inhibiting the translation of mRNA. It was found that the tumor size of *YTHDF1* knockout mice is much smaller than that of wild type, and the immunoreactivity of M6A knockout mice was stronger than that of wild type [41], indicating that *YTHDF1* can be used as an important target of immunotherapy against clinical tumors. In addition, Shi Y et al observed that high expression of *YTHDF1* was associated with better clinical outcomes, and its depletion lead to cancer cells becoming resistant to cisplatin therapy [42]. In our researchers, we found that the increased expression of *YTHDF1* was negatively correlated with risk score, and the down-regulated expression of *YTHDF1* was associated with longer survival in female patients with LUAD, which was consistent with the results of previous studies.

*HNRNPA2B1* plays a role in transcription, mRNA pretreatment, RNA nuclear output, mRNA translation, and mature mRNA stability [43]. Studies have shown that *HNRNPA2B1* was up-regulated by CACNA1G-AS1, thus participating in the progression of NSCLC [44]. *METTL3* participates in the formation of M6A as an encoder. The study found that the OS and progression-free survival of colorectal cancer patients with high expression of M6A regulatory factor *METTL3* were shorter on average [45]. In addition, the expression of *METTL3* is up-regulated in lung cancer, wherein simvastatin treatment can attenuate this increased expression, which can in turn inhibit the malignant progression of lung cancer [46]. Our results also found that the expression of *METTL3* was up-regulated in female LUAD samples, which can also affect the prognosis of the patients. The demethylase *FTO*, as a decoder, can regulate the stability of cellular mRNA by removing M6A residues. Studies have shown that *FTO* can activate the migration of LUAD cells through M6A demethylation and promote the progression of LUAD [47]. The above analysis showed that the five M6A related genes in the risk scoring system were closely related to the occurrence and progression of tumor, which further proved the reliability of the results of this study.

In our study, the expression of *HNRNPC*, *YTHDF1*, *HNRNPA2B1*, and *METTL3* in female LUAD was higher than that in normal tissues, but that of *FTO* was lower. Taking the median risk score as the cut-off value, the risk scoring system could divide female LUAD samples into high-risk group and low-risk group. The overall survival time (OS) of low-risk group was significantly longer than that of high-risk group. The M6A-related risk scoring system developed in this study help to improve the screening of high-risk female patients with LUAD and provides important theoretical bioinformatics support for evaluating the prognosis of such patients. Notably, compared with normal samples, the five M6A-related genes in female LUAD samples were prone to CNV, and have been verified using the sequencing and CNV data in cBioPortal. Further analysis showed a correlation between the copy numbers of *HNRNPC*, *YTHDF1*, *HNRNPA2B1*, *METTL3*, *FTO* and the corresponding gene expression, that is, with an increase in copy number, the expression of these genes increased. In addition, univariate and multivariate analyses combined with clinical factors (such as age and TNM) showed that the performance of the risk scoring system developed in this study in predicting specificity and sensitivity was effective. The M6A-related genes involved in the risk scoring system can be considered new targets in LUAD, and may form the basis for further exploring the pathogenesis and treatment strategies of female LUAD. Although this study may be of vital clinical importance, it undoubtedly has some limitations. More information needs to be collected to verify its accuracy and to conduct further studies *in vivo* and *in vitro* to explore the role and mechanism of action of several M6A-related biomarkers in female LUAD.

## CONCLUSIONS

In this study, we developed a female LUAD risk scoring system based on five M6A-related genes to evaluate the prognosis of patients. The scoring system can classify female LUAD patients into different risk levels according to their expression of these M6A-related genes. In addition, we observed the correlation between the CNVs of five M6A-related genes in the risk scoring system and the corresponding gene expression. We expect that this study will be helpful for early diagnosis and personalized treatment of female LUAD in the future. Although the results of the study need to be further confirmed, the risk scoring system we established may be of great value for the timely prevention of female LUAD and provide important theoretical bioinformatics support for evaluating the prognosis of patients.

## MATERIALS AND METHODS

### Data source

In this study, the mRNA expression, clinical and CNV data used to construct and analysis the female LUAD risk score system were obtained from the TCGA database, and verified by the CNV data of LUAD patients on the cBioPortal platform. We downloaded data related to mRNA expression in 320 female LUADs from the database, including those for 34 normal samples and 286 LUAD samples. Samples without clinical survival data were removed. Finally, data for 266 female LUAD samples were used to develop a risk scoring system. In addition, the CNV data on 623 samples of female LUAD were considered, including those for 323 normal cases and 299 cancer cases. Both TCGA database and the cBioportal platform are open to the public and thus, no further ethical approval was required for this study.

### Data processing and analysis

The level of M6A is dynamically regulated by its encoder (methyltransferase), reader, and decoder. The main genes involved in encoding include *METTL3*, *METTL14*, *WTAP*, *KIAA1429*, *RBM15*, and *RBM15B*. For reader, namely binding protein, the main genes include *YTHDC1*, *YTHDF1*, *YTHDF2*, *YTHDF3*, *HNRNPC*, *HNRNPA2B1*, *FMR1*, and *LRPPRC*. Decoder finger demethylase, and the main genes for decoding include *ALKBH5* and *FTO*. We screened the expression of 16 genes related to M6A from the mRNA expression data of female LUAD. Using the R package of edger software, the expression data of these genes were standardized to elucidate their differential expression in normal samples and female LUAD samples. Then the related genes were looped by FOR sentence, and the violin map was drawn to visualize the results of differential expression. Then, the downloaded CNV data were also analyzed for the normal samples and female LUAD samples.

### Development of a prognostic risk scoring system for M6A-related genes

Although targeted therapy and immunotherapy have greatly improved the outcomes and prognosis of patients with LUAD in recent years, the emergence of drug resistance is still inevitable, and the long-term survival rate is still low. Therefore, it is still an arduous task to evaluate and improve the prognosis of patients with LUAD.

In this study, the construction of female risk scoring system was realized by multivariate Cox analysis. Firstly, 16 M6A related genes were included in the multivariate model, and further screened by multivariate Cox analysis. Finally, the related genes and coefficients that can be used to construct the risk score system were output, and a risk scoring system suitable for evaluating the prognosis of female patients with LUAD was developed. The risk scoring formula is as follows:

$$\text{Risk}\;\text{score}=\sum_{i=1}^{N}Exp_{i}\times \beta_{i}$$

Among the parameters in the above equation, β represents the coefficient of M6A-related genes in the system, and Exp represents the gene expression value. Taking the median risk score as the dividing line, the female LUAD samples in TCGA database were divided into low-risk and high-risk groups to evaluate the prognosis of the patients. Finally, the survival rate of each group was visually displayed through the Kaplan-Meier (KM) curve, and the significance of the risk scoring system for the grouping of female patients with LUAD was intuitively compared.

### Analysis of correlation between risk score and clinical traits

To further evaluate the predictive performance of the risk scoring system that we developed, we included relevant clinical factors in this study. The clinical data of the female patients with LUAD downloaded from TCGA database included age, staging, tumor-node-metastasis (TNM) staging, survival time, and survival status. In order to test the correlation between risk score and clinical characteristics and whether the prognosis of the risk scoring system is independent of other clinical parameters, we included risk score and clinical traits into the variable model, further analyzed the independent prognosis by univariate and multivariate Cox, and further obtained the heat map of the correlation between risk score and clinical traits for visual display. P < 0.05 was considered to indicate statistical significance.

### Correlation and pathway analysis of CNVs in the scoring system

Combined with the CNV data of female LUAD patients in TCGA database, we analyzed the CNVs of M6A-related genes in the risk scoring system, and determined the relationship between CNVs and corresponding gene expression. The expression data and CNV data of five M6A-related genes in the risk scoring system were read in turn by R language, tested and analyzed by ksTest. Then the CNV of related genes was analyzed and verified by using the relevant LUAD data in cBioportal platform. In addition, the KEGG website (https://www.kegg.jp/) was used to analyze the pathway enrichment of M6A-related genes in the scoring system, in order to further understand the possible mechanism of abnormal expression of these genes in female LUADs.
